# Color and brightness at work: Shedding some light on mind wandering

**DOI:** 10.1002/brb3.70020

**Published:** 2024-09-18

**Authors:** Soodabeh Soltanzadeh, Shaghayegh Chitsaz, Reza Kazemi

**Affiliations:** ^1^ Department of Design and Creativity Institute for Cognitive Science Studies Tehran Iran; ^2^ Faculty of Entrepreneurship University of Tehran Tehran Iran

**Keywords:** attention, mind wandering, virtual reality, work environment

## Abstract

**Introduction:**

Occupational hazards are partly caused by the physical factors of the work environment, among which are ambient color and brightness, which can interfere with cognitive performance. Especially in modern work environments, performance relies heavily on cognitive functions such as attention, and an important factor in disrupting sustained attention is mind wandering (MW). This study aimed to investigate the effects of white and blue colors with two brightness levels on sustained attention and brain electrophysiology.

**Methods:**

A total of 20 participants were exposed to 4 different conditions (white and blue as color and 300 and 800 lx as the brightness level) in separate blocks in a virtual reality environment in which a continuous performance test (CPT) was performed.

**Results:**

The high brightness blue condition induced significant changes in sustained attention. MW network analysis showed a significant decrease in delta frequency band in the blue color condition with high brightness and beta decrease in the blue color condition with low brightness, whereas the activity of MW network increased when exposed to the white color condition.

**Conclusion:**

High‐brightness blue light resulted in better sustained attention and decreased activity of MW‐related neural regions. It is thus recommended that these results be taken into consideration in the interior design of educational settings and cars among other environments that require a high level and maintenance of cognitive functions, especially sustained attention.

## Graphical abstract

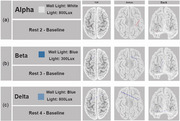

## 1 LIMITATIONS

This study took advantage of a VR environment to simulate work conditions. Although this approach allows for controlled experiments and increasing ecological validity, the extent to which the findings can be applied to real‐world occupational settings is uncertain because the ecological validity of the VR environment may still differ from actual work conditions. Furthermore, this study did not include a control group for comparison that could have facilitated a more comprehensive understanding of the effects of color and brightness on sustained attention and brain electrophysiology. In addition to that, the study primarily focused on sustained attention and MW. Although these are important cognitive functions, other cognitive aspects relevant to occupational performance, such as memory, problem‐solving, and decision‐making, were not assessed here. The authors think that including a broader range of cognitive measures as well as self‐report measures of MW would have provided a more comprehensive evaluation of the impact of color and brightness. Finally, the current study focuses on participants with only university‐level education. This criterion was considered to ensure homogeneity among the participants, due to the known effect of education levels on cognitive function. However, because workplaces have employees with diverse educational backgrounds, future studies should include participants from all educational levels.

## 2 INTRODUCTION

The significantly high prevalence of accidents and injuries in the workplace is obviously one of the main concerns of any employer and employee (Jeong, 2021; Rajak et al., 2022), and one of the predictors of these injuries and accidents is cognitive failure (impairment in memory and attention) (Simpson et al., 2005). On the other hand, optimal job performance heavily relies on the state of cognitive performance while performing tasks at the workplace (Schmitt, 2014; Wright et al., 1995). Attention in general and sustained attention in specific is one of the main cognitive functions required for optimal performance, especially when a high level of vigilance is constantly required, for example, for operators working in an airport control tower (Ribas et al., 2010) and nuclear facilities (Li et al., 2019; Simon & Raghavan, 1993), pilots (Petrilli et al., 2006), and drivers (Edkins & Pollock, 1997; Walker & Trick, 2018). The phenomenon of mind wandering (MW) is one of the main factors disrupting cognitive function in general (McVay & Kane, 2009) and sustained attention in particular (Thomson et al., 2015). MW is a change in the content of thoughts from ongoing work or conflict with events in the external world to self‐generated thoughts (Smallwood & Schooler, 2015) and occurs when the mind wanders away from its primary task and focuses on internal thoughts and unrelated images. Studies have shown that MW is a common phenomenon. For example, students mind wander during lectures 23%–57% of the time (Lindquist & McLean, 2011; Seli et al., 2016; Varao‐Sousa & Kingstone, 2019). Indeed, MW at work has both positive and negative effects. Numerous studies have shown a link between creativity and MW (Fox & Beaty, 2019; Fox & Christoff, 2018; Murray et al., 2021; Yamaoka & Yukawa, 2020). In most jobs, creativity plays a determining role in job function, and MW not only does not have a disruptive role but also serves a constructive and desirable one (Suh & Shin, 2005). However, in some jobs, creativity does not play a determining role, and cognitive functions such as sustained attention are vital. In the present study, we emphasize the negative aspects of this mental phenomenon in the workplace. In addition to that, studies have shown that MW is a common phenomenon in people with attention deficits such as those who suffer from attention‐deficit hyperactivity disorder (ADHD) (Frick et al., 2020). Additionally, recent research has indicated that the dysfunctions in the neural correlates of MW are related to the attentional difficulties of ADHD patients (Bozhilova et al., 2018).

The intersection of neuroscience and environmental psychology aims to investigate the effects of our environment on our psychological and cognitive performance, as well as brain function (Berman et al., 2019, 2021; Tost et al., 2015). Because individuals spend more than 90% of their time inside buildings, indoor environmental quality (IEQ) is crucial for health and well‐being (Jamrozik et al., 2019). IEQ is contingent upon various factors, such as lighting, noise, thermal conditions, and air quality. These factors, whether directly or indirectly, can influence our behavior and productivity (Wang et al., 2021). Consequently, it becomes crucial to comprehend the IEQ factors that yield the most effective outcomes in influencing the behavior and overall satisfaction of the occupants of a space. By mitigating or eliminating the factors that engender discomfort, distraction, or dissatisfaction, it is possible to ameliorate the level of satisfaction experienced by the occupants. Every day, many individuals across the globe experience the repercussions of mishaps and ailments within their respective occupational environments (Ahamed & Mariappan, 2023). According to the International Labor Organization (ILO), it is approximated that on an annual basis, approximately 2.3 million individuals endure fatalities and diseases directly linked to their occupations, with a staggering 6000 individuals succumbing to such circumstances daily worldwide (World Statistics, ILO).

The evidence obtained from workplace health has shown that environmental elements, including indoor air quality, ambient temperature, color, humidity, light, natural views, sound, and dust, are relevant for human health and affect well‐being (Yin et al., 2018). Studies examining the effect of interior design on cognitive performance and psychological state of employees have reported its significant impact on both factors (Karakas & Yildiz, 2020; Llorens‐Gamez et al., 2022; Sharam et al., 2023; Shen et al., 2020). For example, a study examined the effects of a wooden environment on the cognitive function of office employees. In this study, individuals were placed in four different rooms. The rooms varied in terms of the amount of wood and appearance. In one experimental room, the walls, floor, and ceiling were made of white concrete, whereas in two other experimental rooms, the floor, walls, and ceiling were 100% wooden (one room with dark brown wood and the other with light brown wood). In the final room, the materials were 50% concrete and 50% wood. The wooden environment, compared to the non‐wooden environment, led to better reaction times and increased attention (Shen et al., 2020). Another study investigating the cognitive effects of access to daylight and a view showed the positive cognitive effects of access to daylight and view fields (Sharam et al., 2023).

Color is considered a fundamental visual experience for humans, and one of the main components of interior design is color (Gokcakan & Gökçakan, 2016). It acts as a powerful information channel in the human cognitive system (Yang & Jeon, 2020). Individual color preference is associated with an emotional response to the environment and behavior in that environment; therefore, it is necessary to understand how color affects human perception and behavior to create an efficient environment. Various studies have investigated the effect of color on human perception, behavior, and productivity, and there is evidence showing that color affects cognitive and psychological performance (Duyan & Rengin, 2016; Llinares et al., 2021; Min & Lee, 2020). The results of studies on the effects of color on cognitive performance have been contradictory with some showing that blue or red can improve cognitive performance (Llinares et al., 2021), whereas others have found the contrary (Duyan & Rengin, 2016). Previous studies have examined the effects of color on attention and memory in the context of learning (Duyan & Rengin, 2016; Llinares et al., 2021; Llinares Millán et al., 2021; Min & Lee, 2020), and the results indicate that cold colors can increase attention and memory performance (Duyan & Rengin, 2016; Llinares et al., 2021; Min & Lee, 2020). Color plays an undeniable impact on attention, affecting the probability of transferring information to the long‐term memory (Pan & Soto, 2010). Some studies have shown that the effect of color on cognitive performance can depend on the type of the task and level of difficulty. Blue has been shown to improve performance in creative tasks (Mehta & Zhu, 2009) but does not affect the task's difficulty level (Xia et al., 2016).

In another study, the combination of color and shape was examined, which showed that the lower the cognitive load was, the greater the effect of the combination of color and shape on improving cognitive performance turned out to be. The role of color, when combined with shape, is more significant in enhancing cognitive performance (Jin et al., 2022).

One of the reasons for improved attention due to exposure to colors may be the effect of colors on MW. There are studies showing an association between MW and increased activity in the default mode network (DMN) (Kajimura et al., 2016; Poerio et al., 2017). Despite activating areas in the DMN, recent studies have also shown activity in areas, such as dorsal anterior cingulate cortex (dACC), right dorsolateral prefrontal cortex (DLPFC), left ventrolateral prefrontal cortex (VLPFC), secondary somatosensory cortex, left temporopolar cortex, left mid insula, and left lingual gyrus (Fox et al., 2015).

On the other hand, electroencephalography (EEG) studies have shown that the activity of most frequency bands increases during MW in the related neural areas. Previous studies have shown increased activity in delta (Andrillon et al., 2021; Arnau et al., 2020; Braboszcz & Delorme, 2011; Macdonald et al., 2011; Polychroni et al., 2022; van Son, de Rover, et al., 2019), theta (Arnau et al., 2020; Braboszcz & Delorme, 2011; Jin et al., 2019; Kirschner et al., 2012; Polychroni et al., 2022; Rodriguez‐Larios & Alaerts, 2021; van Son, De Blasio, et al., 2019), and alpha frequency bands (Arnau et al., 2020; Baldwin et al., 2017; Boudewyn & Carter, 2018; Compton et al., 2019; Jin et al., 2019; Kam et al., 2021; Macdonald et al., 2011; Polychroni et al., 2022; Wamsley & Summer, 2020), whereas decreased activity has been reported to occur only in beta frequency band (Baird et al., 2011; Cunningham et al., 2000; Martel et al., 2019; van Son, de Rover, et al., 2019). The reported areas related to MW in these studies have been very diverse. In studies that reported an increase in delta power, the electrodes covered the whole head (Andrillon et al., 2021; Arnau et al., 2020), midline central areas (Polychroni et al., 2022), fronto‐central (Braboszcz & Delorme, 2011), and all frontal electrodes (van Son, De Blasio, et al., 2019). In theta, increased activity in all electrodes (Arnau et al., 2020; Rodriguez‐Larios & Alaerts, 2021), occipital parietal regions (Braboszcz & Delorme, 2011; Jin et al., 2019) frontal in both hemispheres and left temporal parietal (Polychroni et al., 2022), and all frontal electrodes (van Son, De Blasio, et al., 2019) have been reported. Moreover, increased alpha has been reported in all electrodes (Arnau et al., 2020), Fz (Baldwin et al., 2017; Boudewyn & Carter, 2018; Wamsley & Summer, 2020), parietal regions (Compton et al., 2019), occipital parietal areas (Jin et al., 2019; Macdonald et al., 2011), Oz (Kam et al., 2021), and all frontal electrodes (Polychroni et al., 2022). Finally, decreased beta has been reported in the frontal and parietal (Baird et al., 2011), occipital parietal (Martel et al., 2019), occipital (Braboszcz & Delorme, 2011), parietal (Cunningham et al., 2000), and all frontal electrodes (van Son, De Blasio, et al., 2019).

Due to the lack of consensus regarding the brain regions during MW in previous EEG studies, the use of classical approaches such as power analysis and examining connectivity such as coherence can be problematic. Therefore, the lack of consensus has brought attention to functional connectivity (FC) analysis in resting state EEG (Krukow & Jonak, 2022). In this study that investigated the relationship between neural activity in the DMN and MW, the results show that there is a correlation between scores in MW and the activity of four frequency bands, namely delta, theta, alpha, and beta (Krukow & Jonak, 2022). Furthermore, studies that took advantage of concurrent use of EEG and fMRI have been able to validate the previous FC findings. For example, the result of a recent study shows that during MW, an increase in delta, theta, and alpha activities and a decrease in beta occur, which is exactly consistent with the results of the studies discussed above (Groot et al., 2021). Therefore, in the present study, due to the lack of consensus in the studies conducted at the sensor level regarding the specific regions of activity, FC analysis in neural networks related to MW has been used.

The aim of the current study was to investigate the combined role of color and brightness on MW and task performance. The present study was designed to understand the effects of color on the activity of the neural regions related to MW and improving the attentional performance in the workplace using a virtual reality (VR) experiment.

## 3 MATERIALS AND METHODS

### 3.1 Participants

A total of 28 people expressed their willingness to participate in the experiment after seeing the advertisement of the study on social media in September 2022. The sample size required for the current study was calculated utilizing G*Power software version 3.1.9.2 (Franz Faul; University of Kiel, Kiel, Germany). The results demonstrated that 20 participants would provide a statistical power of .85 and an effect size of .40 for the multivariate analysis of variance (MANOVA). In addition, observed power was calculated through SPSS (Statistical Package for Social Science, observed power = .92). On the basis of the power analysis, 20 people (10 women and 10 men) with an average age of 29.6 were selected based on the inclusion and exclusion criteria. The inclusion criteria were as follows: age between 18 and 40 years, having a university‐level education, being right‐handed, and completing a written consent form. The exclusion criteria were as follows: experiencing psychiatric disorders, being diagnosed with neurological diseases, and unwillingness to continue participating in the study. The conduct of this research was approved by the Ethics Committee of the Institute for Cognitive Sciences Studies (ICSS) (IR.UT.PSYEDU.REC.1399.009).

### 3.2 Study design

**FIGURE 1:**
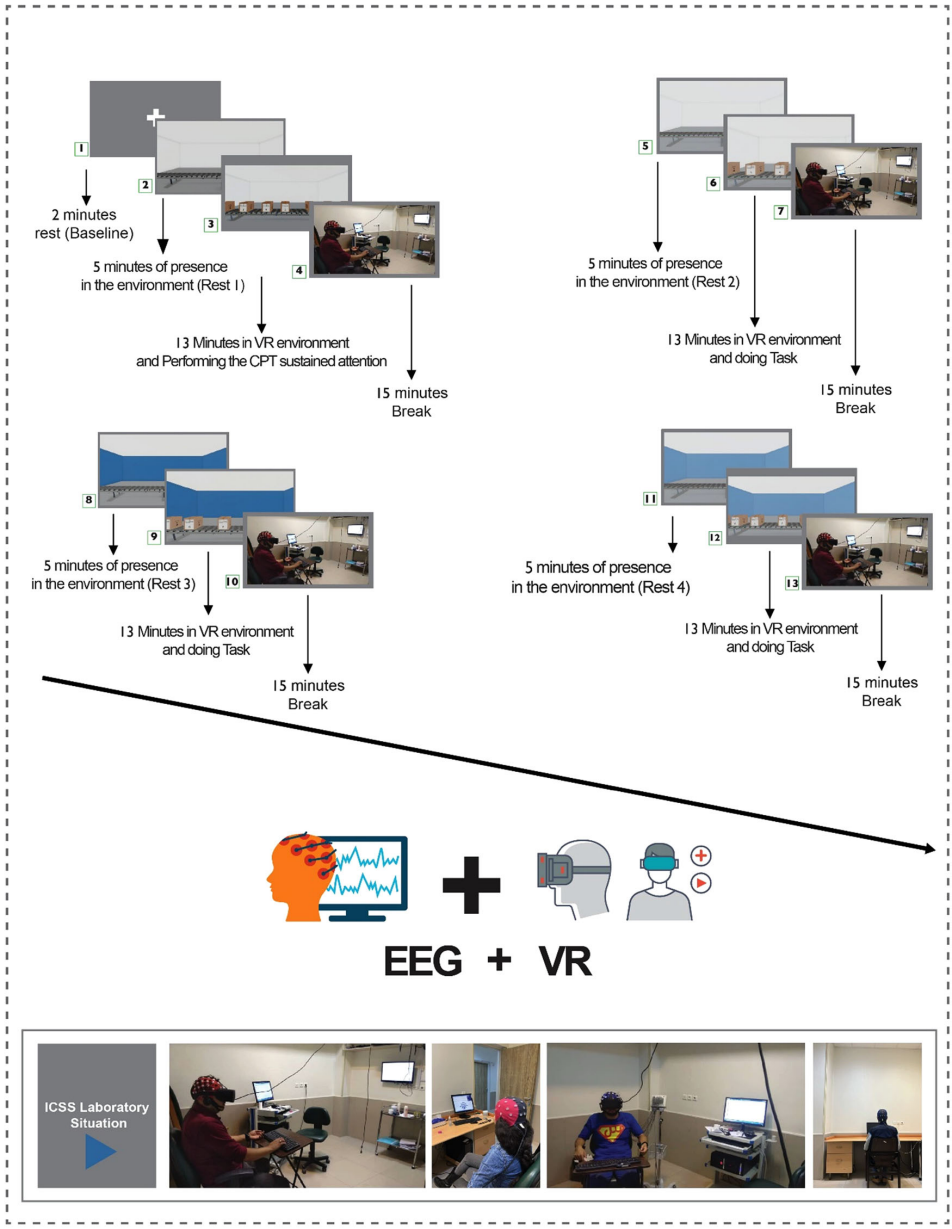
All subjects had electroencephalographies (EEGs) recorded with their eyes closed before entering the virtual reality (VR) environment (baseline). Subjects then put on the VR glasses and remained in the VR environment without performing any tasks (Rest). After that, subjects performed the continuous performance test (CPT) task for 13 min. The VR environment comprised four different conditions of white and blue colors with two levels of illumination (300 and 800 lx). Each subject was exposed to all four conditions in a random manner.

The experiment was conducted from August to October 2022 at the ICSS, Tehran, Iran. Each participant received relevant instructions (Supporting Information) prior to the start of the experiment. Eyes‐closed EEG was first recorded for 2 min before the VR glasses (Oculus VR) were put on. This EEG recording that was acquired outside the VR environment will be referred to, throughout the manuscript, as baseline and is used to compare and contrast brain activity during exposure to the experimental conditions. In the VR context, participants were first placed in the workspace position for 5 min, and then performed the VR version of the continuous performance test (CPT) for 13 min. The 5‐min VR immersion, in which participants did not engage in any activity or task other than just being in a specific environment, was naturally repeated four times corresponding to the four combinations of color and light intensity. These four combinations are presented in Table 1. The EEG recording corresponding to each of these conditions will be referred to as Rest 1, Rest 2, and so on, meaning that the EEG was recorded when the participant was in the VR environment corresponding to the number of conditions. Worth noting is that the word Rest is used because participants during these intervals do not engage in task performance, although they are exposed to the experimental condition in the VR environment. For example, Rest 1 refers to the EEG data corresponding to the first condition, that is, when the brightness level was set at 300 lx and the ambient color was white. The orders of the presentation of these conditions were random among participants. The experimental procedure is shown in Figure 1. After completing the CPT task, to relieve fatigue and prevent side effects of engaging with a virtual environment, the VR glasses were removed. After a brief instruction and 15 min of break, the test procedures were continued, and participants were exposed to the remaining conditions. The experiment lasted for almost 3 h.

**TABLE 1:** Details of the virtual reality (VR) experimental conditions.

Condition	Brightness (lx)	Color
1	300	White
2	800	White
3	300	Blue
4	800	Blue

### 3.3 Cognitive task

CPT is used to assess sustained attention, that is, the ability to maintain focus over time on a given task (Ghassemi et al., 2009). CPT paradigms generally involve multiple repetitions of rapid presentations of stimuli with infrequently occurring targets (Gaume et al., 2019). In the present study, the task was designed based on the CPT in the VR context. In this test, two types of omission and commission errors are scored. An omission error occurs when the participant fails to respond to the target stimulus and indicates that the individual has difficulty understanding the stimulus. This type of error is interpreted as a problem in the stability of attention and indicates inattention to the stimuli. An error of commission occurs when the participant responds to a nontarget stimulus showing a weakness in inhibition and is interpreted as a problem in impulse control or impulsivity. In addition to these two errors, the number of correct answers and the reaction time of people to the stimulus were also calculated.

**FIGURE 2:**
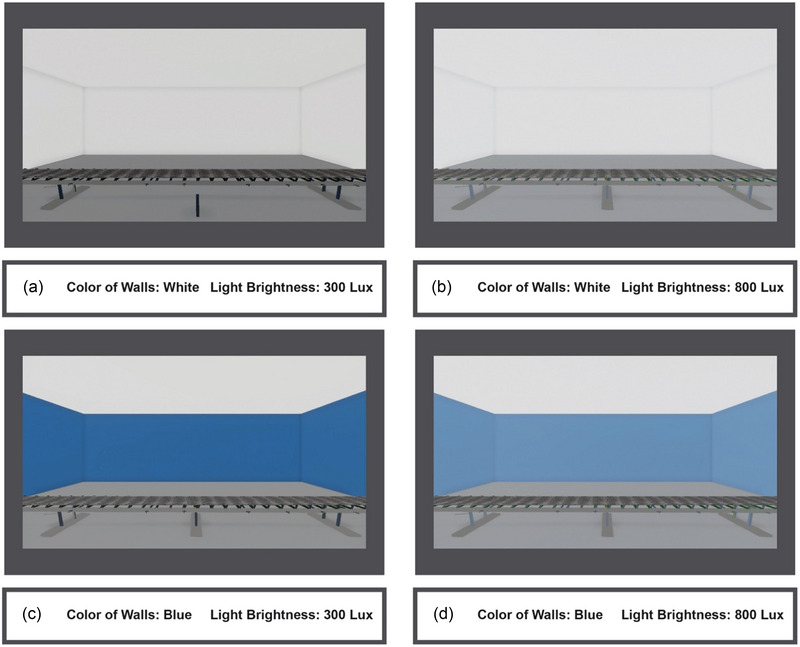
The virtual reality (VR) environment included four different combinations of color and brightness: white wall and 300 lx brightness (a), white wall and 800 lx brightness (b), blue wall and 300 lx brightness (c), and blue wall and 800 lx brightness (d).

The VR task was designed to simulate the actual work environment in which packages moved on the conveyor belt in front of the participants. Single‐digit numbers from “0” to “9” were written on the label of each of these boxes, and the participants were required to identify the boxes with the target stimulus, considered the number “0” in this study. In this study, to control the participants’ movement and record EEG waves with less noise, participants were asked to press the button whenever the number “0” appeared on the boxes in the VR environment. Click “Enter” on the keyboard with the dominant hand, and do not answer if they saw other digits. In addition, participants were also asked to remain still during the experiment to reduce noise (Figure 2).

The numbers on the boxes were randomly selected from single digits (0–9), and the time interval between the appearances of each box (stimuli) was a random number between 2000 and 1500 ms, with the maximum time allowed to respond to the target stimulus being 1500 ms (Min et al., 2013). The target and the nontarget stimuli were presented with frequencies of 30% and 70%, respectively. The number of correctly identified cases of the target stimulus was considered the test score in addition to registering the number of items that were incorrectly selected, the number of missed targets, and the reaction time.

### 3.4 Virtual reality environment design

**FIGURE 3:**
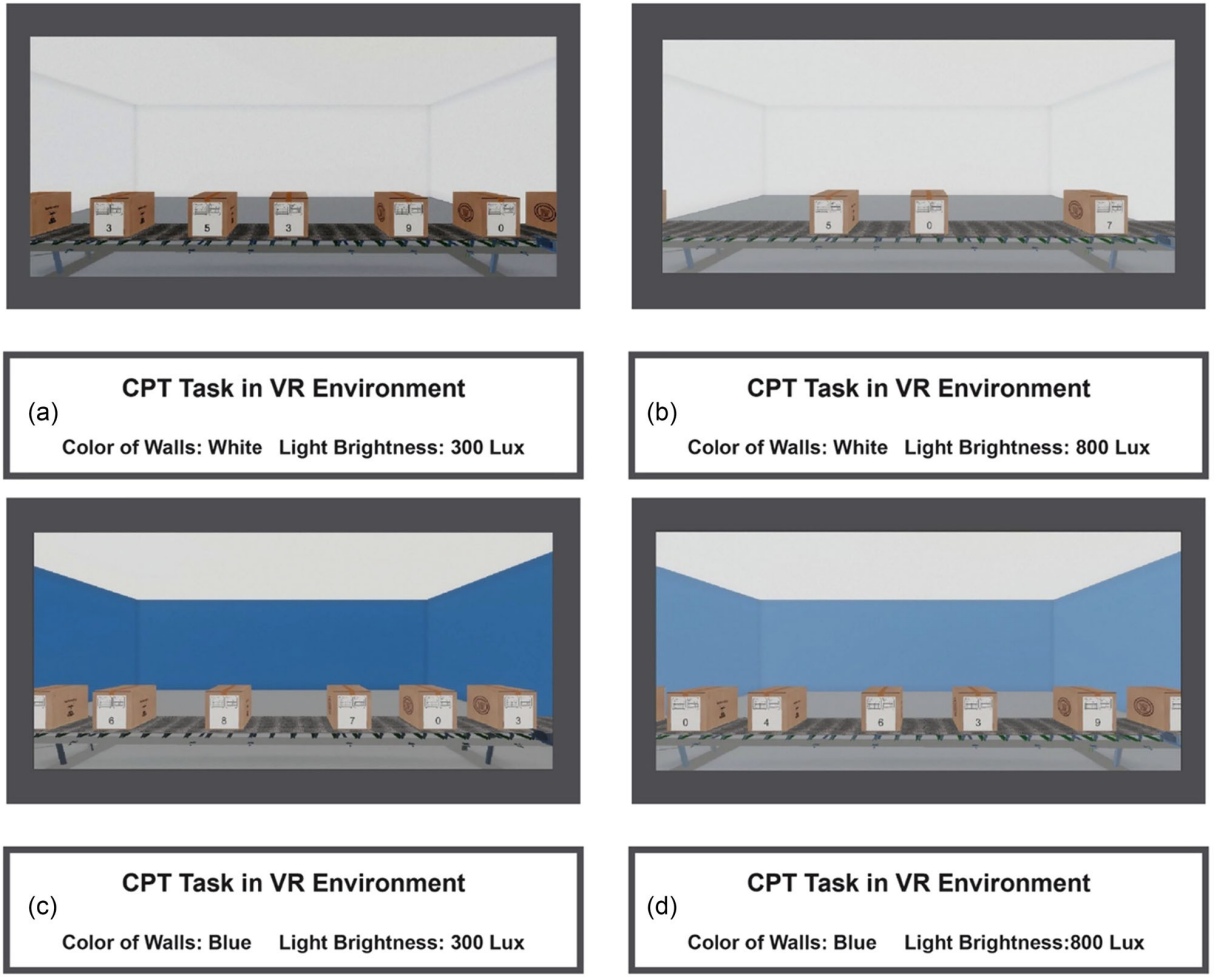
In the virtual reality (VR) environment, the participants could see boxes with numbers between 0 and 9 on them and had to press the ENTER button on the keyboard when they saw the box with zero on it. The interval between each box presentation was random and between 1500 and 2000 ms, and the time window for responding to the target stimulus was 1500 ms.

The simulated environment in this research is an HDRI model in version 2020.3.5F with the ability to adjust the natural environment light. The design of this model was done using the UNITY software (Unity Technologies). A simulated workspace is a windowless room with artificial lighting. The color of the floor was gray, the color of the ceiling was white, and the color of the walls was white in Conditions 1 and 2 and blue in Conditions 3 and 4. Moreover, the intensity of lighting was set at two values of 300 and 800 lx according to the recommended standards for the appropriate lighting intensity in the workspace and the level that may potentially affect cognitive functions (Bao et al., 2021). Figure 3 shows the simulated environmental scenarios. A background noise associated with a regular workspace was also considered in the VR environment to make the environmental circumstances as realistic as possible. Considering that other investigations recommend a minimum presence of 5 min in the environment for the effect of the physical factors in the space to take place (Barton & Pretty, 2010; Van den Berg et al., 2015), participant exposure time to each simulated situation included 5 min of being in the environment at rest and 13 min of performing the task, making a total of 18 min.

### 3.5 EEG data recording and preprocessing

EEG data were recorded using a Q5000 EEG device (Negar Andishgan Company). Ag/AgCl electrodes were used to record EEG signals installed on an elastic cap. A total of 32 electrodes based on the International 10‐20 system were placed on the head. The EEG electrodes included FP1, FPZ, FP2, F7, F3, FZ, F4, F8, FC5, FC1, FCZ, FC2, FC6, T7, C3, CZ, C4, T8, CP5, CP1, CPZ, CP2, CP6, P7, P3, PZ, P4, P8, POZ, O1, OZ, and O2. The impedance between the electrodes and the skin was kept below 5 kΩ using conductive gel. The sampling rate in this study was 500 Hz, and the reference electrode was CZ with the GROUND electrode placed on the wrist. The EEG data were preprocessed using the EEGLAB toolbox (Delorme & Makeig, 2004; Hatz et al., 2015) running on MATLAB (The MathWorks, Inc.) to eliminate artifacts, following a visually controlled protocol (Hatz et al., 2015). The data were initially filtered using a finite impulse response high‐pass filter with a cutoff frequency of 0.5 Hz and a low‐pass filter with a cutoff frequency of 40 Hz, both applied with zero‐phase shift. Moreover, a 50 Hz notch filter was used to remove the power line noise. After removing bad channels using the FASTER plugin (Nolan et al., 2010), any artifactual time points, including those caused by body movements, were detected and removed through visual inspection. Then, an independent component analysis (ICA) was used with the logistic Infomax algorithm (Langlois et al., 2010) to detect and remove nonneural artifacts, such as electrooculograms and cardiac artifacts. Additionally, the multiple artifact rejection algorithm (Winkler et al., 2011) was applied to enhance the artifact rejection process by facilitating the identification of artifactual components. Subsequently, the removed EEG channels were interpolated using a spherical spline interpolation method (Perrin et al., 1989). Finally, the continuous EEG data were segmented into 60‐s nonoverlapping epochs for further analysis.

### 3.6 EEG analysis

#### 3.6.1 Source localization

eLORETA enables the calculation of the cortical distribution of current density. eLORETA has improved significantly compared to previous tomographies of LORETA and sLORETA. In this method, there is no spatial bias in localization in the presence of structural noise. Localizing the current source accurately in deep areas of the brain is possible. The head model for eLORETA and the coordinates of the electrodes are based on the Montreal Neurological Institute (MNI) coordinates. In eLORETA, the spatial solution is limited to the gray matter according to 6239 voxels of 5 × 5 × 5 mm^3^. eLORETA tomography has been validated in several fMRI (Vitacco et al., 2002), structural MRI (Worrell et al., 2000), and PET (Dierks et al., 2000) studies.

Artifact‐free EEG segments were used to calculate the eLORETA intracranial spectral density with a 1 Hz resolution from 0.5 to 45 Hz. eLORETA functional images of spectral density were calculated for the following eight frequency bands: delta (0.5–4 Hz), theta (4–8 Hz), alpha1 (8–10.5 Hz), alpha2 (10.5–13 Hz), beta1 (13–18 Hz), beta2 (18–30 Hz), beta3 (13–30 Hz), and gamma (30–40 Hz).

#### 3.6.2 Functional connectivity analysis

A voxel‐wise approach was considered to determine the regions of interest (ROIs) in the FC analysis. To generate the ROIs, MNI coordinates of the areas under every electrode were specified by eLORETA. Based on previous studies on MW networks, 13 ROIs were defined as (Fox et al., 2015) presented in Table 2.

**TABLE 2:** List of regions of interest (ROIs) used in the current study and Montreal Neurological Institute (MNI) coordinates.

MNI coordinates			Lobe (hemisphere)	Region	BA
*X*	*Y*	*Z*			
−5	27	39	Frontal lobe	Dorsal anterior cingulate cortex	3
45	43	−8	Frontal lobe (R)	Dorsolateral/Rostrolateral prefrontal cortex	46/10
−35	10	−26	Frontal lobe (L)	Ventrolateral prefrontal cortex	47/11
3	61	13	Frontal lobe	Rostromedial prefrontal cortex	10/9
4	42	3	Frontal lobe	Medial prefrontal cortex; anterior cingulate cortex	24/32
−8	−56	39	Parietal lobe	Precuneus; posterior cingulate cortex	7/31
−46	−72	25	Parietal lobe (L)	Inferior parietal lobule; angular gyrus	39
56	−51	33	Parietal lobe (R)	Inferior parietal lobule; supramarginal gyrus	40/39
24	−39	56	Parietal lobe (R)	Secondary somatosensory cortex	5/40
−27	−37	−18	Temporal lobe (L)	Parahippocampus	36
−50	−1	−5	Temporal lobe (L)	Temporopolar cortex	38
−42	29	−12	Temporal lobe (L)	Mid‐insula	13
−15	−66	5	Occipital lobe (L)	Lingual gyrus	19/18/30

Lagged phase synchronization was used to analyze the FC among all the ROI pairs. This method assesses the similarities among signals in the frequency domain based on the normalized Fourier series. Lagged connectivity is an accurate and corrected index demonstrating the relations between two signals after omitting the instantaneous zero‐lag component. This correction is essential when working with scalp or intracranial EEG signals because zero‐lag connectivity in a specific frequency band is mainly due to the non‐physiologic effects or intrinsic physical artifacts, especially volume conductivity and low spatial resolution.

The details of its calculation are elaborated by Pascual‐Marqui et al. (2011) and Canuet et al. (2011). However, it will be briefly mentioned here by considering the following equation:

(1) ϕ2x,yω=Imfx,yω21−Refx,yω2

where x and y refer to the discrete Fourier transform of the two x and y signals, and ω signifies the bandwidth. Re[c] and Im[c] refer to the real and imaginary components of the complex number c. Instantaneous connectivity (zero‐lag) is related to the fundamental component of phase synchronization.

### 3.7 Statistical analysis

The SPSS software version 23 was used for statistical analysis in this study. A value of *p* < .05 was considered the significance level. MANOVA was used to examine the relationship among different situations (white light, intensity 300 lx/white light, intensity 800 lx/blue light, intensity 300 lx and blue light, intensity 800 lx) with sustained attention performance (accuracy, attention, inhibition, and reaction time). FC analysis in all the possible pairs and eight frequency bands was done using eLORETA. To compute FC between the baseline (resting state) and VR environment in each frequency band, paired *t*‐tests and randomization were used. In addition, to correct for multiple comparisons, nonparametric randomization based on maximum statistics was used in eLORETA.

## 4 RESULTS

### 4.1 Cognitive task

CPT task was performed to assess the potential differences in the dependent variables (accuracy, attention, inhibition, and reaction time) among the four conditions (Table 1). Descriptive statistics of the CPT indices, namely, accuracy, attention, inhibition, and reaction time for each of the conditions, are presented in Table 3.

**TABLE 3:** Descriptive statistics of the continuous performance test (CPT) indices in different conditions.

Condition		*N*	Minimum	Maximum	Mean	Std. deviation
1. 300 lx white	Accuracy	20	29.00	276.00	139.50	66.54
	Attention	20	124.00	371.00	250.55	75.75
	Inhibition	20	90.00	364.00	233.80	84.24
	Reaction time	20	.01	1.31	.45	.49
		20				
2. 800 lx White	Accuracy	20	40.00	349.00	159.75	93.70
	Attention	20	51.00	358.00	239.45	92.36
	Inhibition	20	20.00	349.00	189.80	111.57
	Reaction time	20	.01	1.10	.31	.35
		20				
3. 300 lx blue	Accuracy	20	77.00	356.00	205.55	87.32
	Attention	20	43.00	323.00	189.60	89.11
	Inhibition	20	33.00	310.00	177.65	88.71
	Reaction time	20	.00	1.22	.44	.39
		20				
4. 800 lx blue	Accuracy	20	48.00	379.00	232.90	90.79
	Attention	20	22.00	352.00	178.35	93.24
	Inhibition	20	13.00	323.00	163.20	88.74
	Reaction time	20	.00	1.22	.42	.41
						

The MANOVA showed that there is a significant difference in at least one of the dependent variables (*F*(12,193) = 2.353, *p* < .05, Eta = .11). The results of MANOVA showed that conditions have a significant effect on “attention” (*F*(3,76) = 3.321, *p* = .02, Eta = .11) and “accuracy” (*F*(3,76) = 4.973, *p* < .003, Eta = .16), but no significant changes were observed in “inhibition” (*F*(3,76) = 2.104, *p* > .05, Eta = .07) and “reaction time” (*F*(3,76) = .487, *p* > .05, Eta = .01).

**FIGURE 4:**
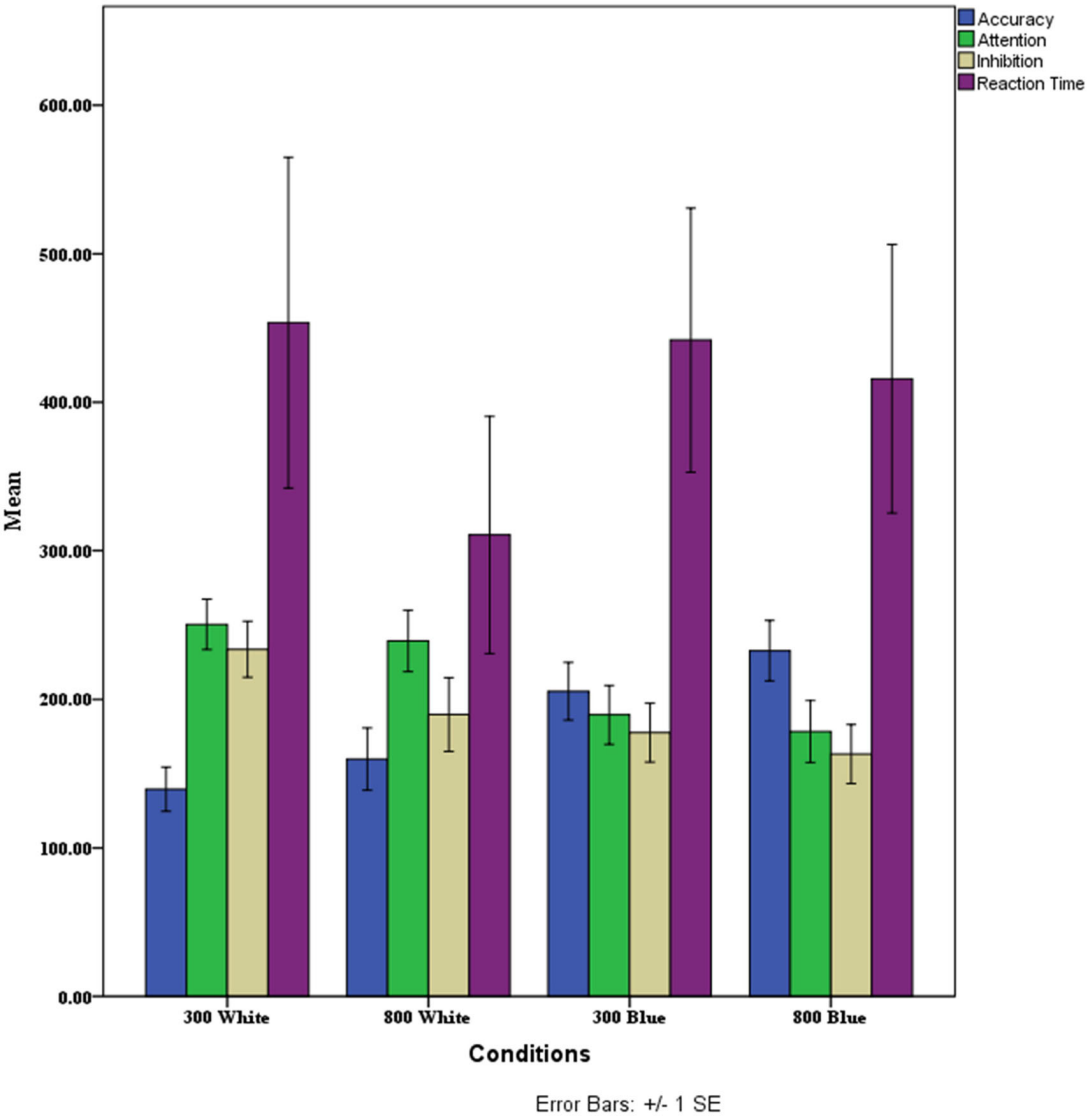
Mean values of accuracy, attention, inhibition, and reaction time across the four experimental conditions. *Note*: Reaction times are presented in milliseconds.

Tukey's post hoc test results showed a significant difference between Conditions 1 and 4 (*p* = .005) and 2 and 4 (*p* = .04), and a marginally significant difference between Conditions 1 and 3 (*p* = .07) in “accuracy”; a significant difference between Conditions 1 and 4 (*p* = .05) in “attention”; and a marginally significant difference between Conditions 1 and 4 (*p* = .09) in “inhibition.” Figure 4 shows how these measures compare and contrast across different experimental conditions.

### 4.2 EEG functional connectivity

**FIGURE 5:**
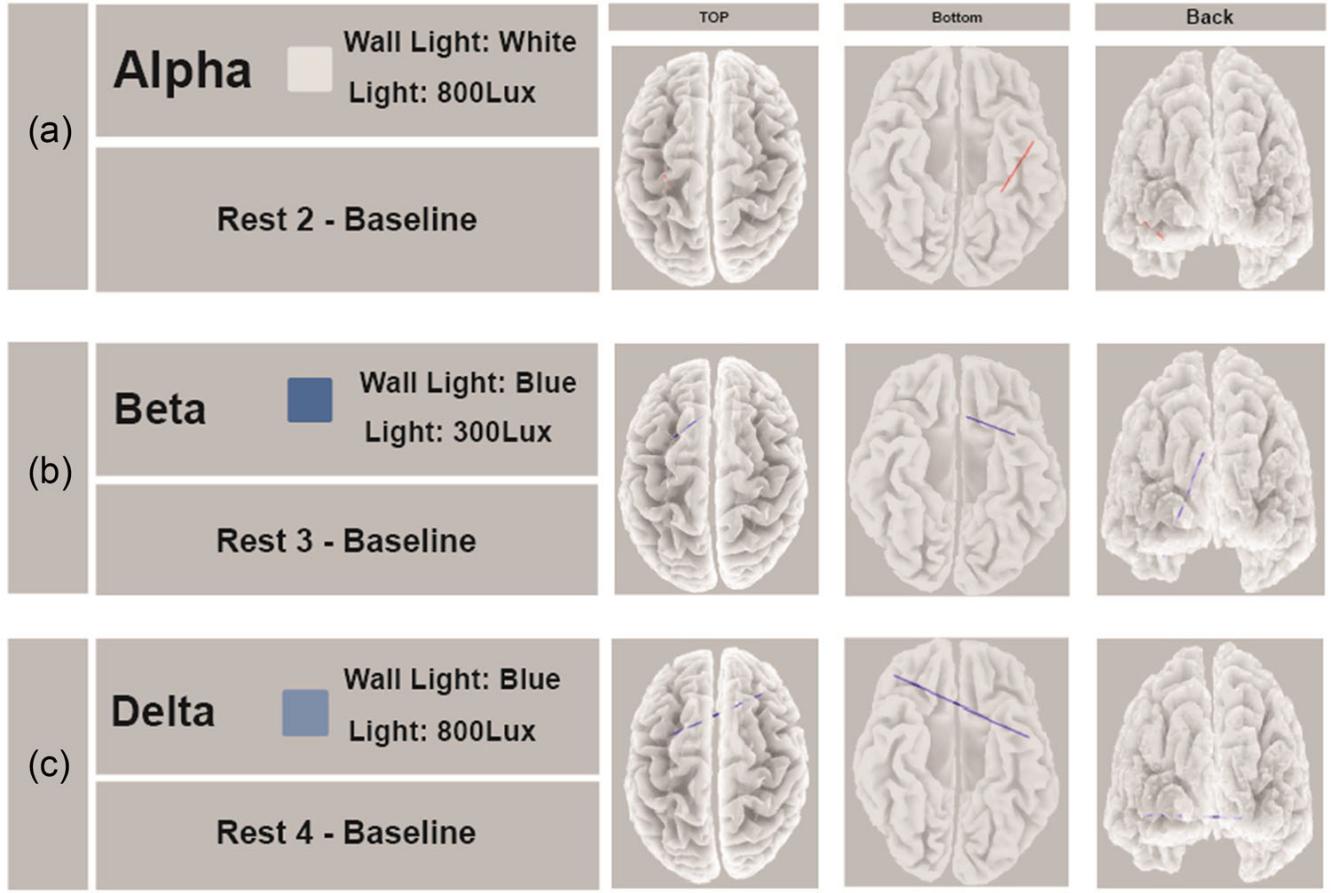
eLORETA wire diagram shows the cortical areas that had a significant decrease and increase in lagged phase synchronization. Comparing Condition 2 with the baseline showed that lagged phase synchronization between left parahippocampus and left temporopolar cortex had a significant increase in alpha frequency band (a). Comparing Condition 3 with the baseline showed that lagged phase synchronization between dorsal anterior cingulate cortex and left ventrolateral prefrontal cortex in the beta frequency band was decreased (b), and comparing Condition 4 with the baseline showed a significant decrease in lagged phase synchronization between right dorsolateral/rostrolateral prefrontal cortex and left Temporopolar cortex in delta frequency band (c).

The results of the FC analysis showed a significant increase in lagged phase synchronization in the alpha frequency band between left parahippocampus and left temporopolar cortex (*t*_max_ = 3.55, *p *< .02) (Figure 5a). A significant decrease in lagged phase synchronization between dACC and left VLPFC in beta frequency band (*t*_max_ = −4.38, *p *< .02) was observed in Rest 3—baseline (Figure 5b). Moreover, a significant decrease in lagged phase synchronization between right DLPFC/rostrolateral prefrontal cortex and left temporopolar cortex in delta frequency band (*t*_max_ = −3.18, *p *< .03) was observed in Rest 4—baseline (Figure 5c).

## 5 DISCUSSION

The results of our study showed that the blue color with high brightness can improve sustained attention performance and reduce the activity of neural regions related to MW (frontal/temporal). High brightness white color increased the activity of neural regions associated with MW (temporal) and did not result in any improvement of sustained attention. The best condition for improving sustained attention both behaviorally and neurally was blue color with a high brightness.

Regarding the effect of the blue color on cognitive performance, the results of the studies so far have been contradictory, with some studies reporting improvements in attention (Llinares et al., 2021) and others deterioration (AL‐Ayash et al., 2016). A positive and not negative effect (Ainsworth et al., 1993; von Castell et al., 2018) has been reported as a result of exposure to blue or a *cold* color. Studies using electrophysiological tools have shown that exposure to the blue color increases activity in theta, alpha (Bower et al., 2022), and beta (Bower et al., 2022; Llinares Millán et al., 2021) frequency bands. Moreover, the use of blue color in the building increases the range of autonomic (sympathetic) activity, which is related to the modulation of brain activity in emotion processing.

Blue color with low brightness in our study did not cause a significant change neither in the sustained attention performance nor the MW network; however, it caused a significant decrease in connectivity in the beta frequency between the dACC and the left VLPFC. Previous studies showed that MW decreases beta power (Groot et al., 2021) and decreases connectivity (Krukow & Jonak, 2022) in areas related to MW, with theta‐to‐beta ratio as one of the valid indices frequently used in MW studies (van Son, De Blasio, et al., 2019; van Son, de Rover, et al., 2019). The poor performance of the participants in the sustained attention test when exposed to blue color with low brightness can be caused by the increase in the activity of the nervous areas related to the emotional system, which is the leading cause of this increase in the activity of the MW‐related neural areas. Exposure to blue can increase MW, and the start of MW can increase autonomic (sympathetic) activity and decrease sustained attention during the activation of the MW network, which overlaps a lot with the DMN; the areas related to the task‐positive network decrease in activity, which can lead to difficulty in performing cognitive tasks. On the other hand, studies have shown that a decrease in MW is associated with an increase in heart rate variability, and an inverse relationship has been reported between them (Bortolla et al., 2022).

Although exposure to blue color with low brightness did not result in changes in sustained attention, blue color with high brightness improved the performance of sustained attention and decreased neural connections in MW network in delta frequency band between left DLPFC/rostrolateral prefrontal cortex and left temporopolar cortex. All negative changes were reversed with increasing brightness. Different studies have shown that the combination of color and brightness can cause changes in brain electrophysiology (Cornelissen et al., 2006; Park et al., 2013; Xing et al., 2015). Colors with more brightness are associated with positive emotions, and colors with less brightness are associated with negative emotions (Takahashi & Kawabata, 2018). A recently published study (Eroğlu et al., 2020) has shown different brightness levels have a differential effect on emotional processing and EEG power. Increasing the brightness of pleasant and neutral images increases the EEG power in the posterior regions (parietal and occipital), whereas increasing the brightness of unpleasant images decreases the EEG power in these regions. Moreover, increasing the brightness reduces the arousal caused by unpleasant images. Various studies have shown that the delta frequency band is related to emotional processing (Klados et al., 2009; Knyazev et al., 2009). The delta frequency band increases under the influence of the arousing emotional stimulus component in the posterior regions (Knyazev et al., 2009), and delta synchronization is also affected by the emotional stimulus (Klados et al., 2009). A study investigating the delta response affected by exposure to emotional images with high brightness (Kurt et al., 2017) found that unpleasant emotional images with high brightness caused a lower delta amplitude than the same images with normal brightness. In general, it can be concluded that blue color with more brightness can increase positive emotion and subsequently decrease MW and improve sustained attention.

Previous research indicates the negative impact of the white color on cognitive performance (Grangaard, 1995; Kwallek et al., 1996; Niero & Premier, 2010). In our study, white color did not affect the performance of sustained attention and increased the activity of MW‐related regions. The effect of white color with high brightness on the neural areas of MW can have an effect on the proper engagement of task‐related areas of the brain, and disruption of this activity can lead to poor performance in sustained attention.

## 6 CONCLUSION

This study investigated the effect of color on MW and showed that blue color with high brightness improves sustained attention performance by reducing the activity in the MW‐related neural areas. MW is a significant factor in work injuries and traffic accidents, and the use of blue color with optimal lighting in the interior design of buildings and cars can be a preventive factor for accidents that may completely or partially be a result of MW. While driving, for example, ambient lighting systems typically remain illuminated (Flannagan & Devonshire, 2012), and their purpose is to facilitate the driver's ability to perceive and manipulate the interior controls and enhance the driver's emotional state, attentiveness, and overall comfort. The significance of ambient lighting lies in its ability to provide a heightened sense of direction, an improved perception of spaciousness, and a feeling of safety, value, and comfort, which are all relevant to a vast range of work and daily life activities. Overall, the findings of the current study provide further evidence regarding the impact of color and light intensity on a measure of attention and its potential impact on safety, productivity, and comfort either in a vehicle or any other work environment. More systematic investigations are required to validate these findings and further our understanding of the impact of light and brightness on our cognitive capacities and how they influence our behavior and experience.

## AUTHOR CONTRIBUTIONS

**Soodabeh Soltanzadeh**: Conceptualization; project administration; formal analysis; data curation; writing—review and editing. **Shaghayegh Chitsaz**: Conceptualization; methodology; writing—review and editing. **Reza Kazemi**: Conceptualization; methodology; formal analysis; writing—original draft.

## CONFLICT OF INTEREST STATEMENT

The authors declare no conflicts of interest.

## FUNDING INFORMATION

This research received no specific grant from any funding agency in the public, commercial, or not‐for‐profit sectors.

### PEER REVIEW

The peer review history for this article is available at https://publons.com/publon/10.1002/brb3.70020.

## CONSENT

In accordance with the Declaration of Helsinki, all participants gave their written informed consent to participate in the research.

## Supporting information

Supporting Information

## Data Availability

The data that support the findings of this study are openly available in Figshare at https://figshare.com/, reference number http://doi.org/10.6084/m9.figshare.26342269.
