# Supplementary material for: Color and brightness at work: Shedding some light on mind wandering
Source: Brain Behav. 2024 Sep 18;14(9):e70020. doi: 10.1002/brb3.70020 (PMC11410860; doi:10.1002/brb3.70020)
Supplement: Supplementary file 1 — Supporting Information [file BRB3-14-e70020-s001.docx]

S1.

Before entering the virtual reality environment, it was explained to the subjects in detail that in each of the four scenarios of this study, they will first rest for 5 minutes in the designed virtual environment, and from the 6th minute, At the start of the task, a conveyor belt appears in the virtual room, on which boxes move, each of which has single-digit numbers from "0 to 9" written on them, and they must be identified. Boxes with the number "0" act, and by pressing the "ENTER" button on the keyboard with the dominant hand, answer it and do not give any answer if you see other numbers. In addition, the participants were told that the task lasted for 13 minutes. Then, with a 15-minute break, the test procedures were repeated, and people faced the following scenario and performed the task. During the experiment, people were asked to avoid blinking, moving, and talking to reduce the noise.
